# Detection of COVID-19 from CT Lung Scans Using Transfer Learning

**DOI:** 10.1155/2021/5527923

**Published:** 2021-04-08

**Authors:** Sahil Lawton, Serestina Viriri

**Affiliations:** School of Mathematics, Statistics and Computer Science University of KwaZulu-Natal, Durban, South Africa

## Abstract

This paper aims to investigate the use of transfer learning architectures in the detection of COVID-19 from CT lung scans. The study evaluates the performances of various transfer learning architectures, as well as the effects of the standard Histogram Equalization and Contrast Limited Adaptive Histogram Equalization. The findings of this study suggest that transfer learning-based frameworks are an alternative to the contemporary methods used to detect the presence of the virus in patients. The highest performing model, the VGG-19 implemented with the Contrast Limited Adaptive Histogram Equalization, on a SARS-CoV-2 dataset, achieved an accuracy and recall of 95.75% and 97.13%, respectively.

## 1. Introduction

The COVID-19 pandemic has had a major impact on the world, with over 55 326 907 confirmed cases and 1 333 742 confirmed deaths (according to the World Health Organization) as of the 19th of November 2020. Contraction of the virus often results in a respiratory disease, in which common symptoms include fever, coughing, sore throat, short breathing, headache, and diarrhea. There are new vaccines, some at trial and initial stages to cure the virus, and therefore the use of social distancing and rapid mass testing has been resorted to, in an attempt to minimize its impact.

One of the recently developed testing methods, the serology test, has become the gold standard in testing and shows impressive performances, with a recall of 70%–85% between 5 and 10 days after infection, and 85%–90% between 10 and 14 days after infection. This test, however, has taken months to develop and replaces the previously used RT-PCR test, which not only shows a poor recall of 71% [1] but also has an added disadvantage of being a completely manual and time-consuming process, with results taking up to two days.

An alternative approach to detecting the virus can be found in use of radiological images such as CT scans. The presence of features in the scans such as bilateral and peripheral predominant ground-glass opacities can indicate early stage of infection, while air space consolidation often correlates with the peak stage of infection. Advancements in computer vision can also allow for the development of tools to help create an automated process of diagnosing the disease from these images and, in doing so, allow for another line of testing, which could assist in the global fight against this pandemic. In this paper, an investigation is conducted on the use of transfer learning in the task of detecting COVID-19 from lung scans. Transfer learning is a strategy wherein the knowledge mined by a machine learning algorithm from one set of data is transferred to solve a different but related task, involving new data, wherein the volume of data available for the new task may be limited [2].

In this study, the knowledge gained by training a series of different convolutional neural networks (CNN) on a large scale, hierarchical dataset called ImageNet [3], is transferred to the task of detecting COVID-19 from CT lung scans.

The series of pretrained CNN's are retrained on the COVID-19 lung scan dataset, and the resulting models are evaluated to determine the best model for the development of a framework for COVID-19 detection. Given the reliance of deep learning models on a large quantity of data for optimal training, the transfer of knowledge from a large-scale generic dataset such as the ImageNet database to a specific COVID-19 dataset can allow for the development of high performing models that compensate for the limitations in training data for the latter. This approach is useful when highly accurate models need to be developed to tackle “Black Swan” events, such as the emergence of a new virus, and the requirements of mass testing. The use of deep convolutional networks also allows the automatic mass feature extraction. The study evaluates and compares the performance of five different transfer learning models, namely, the DenseNet-201 [4], ResNet-101 [5], MobileNet-V2 [6], EfficientNet-B4 [7], and VGG-19 [8], which, according to the literature [9–12], have shown to be amongst the best performing architectures in detecting the presence of COVID-19 from lung scans.

Furthermore, this study investigates the effect of standard Histogram Equalization (HE) and Contrast Limited Adaptive Histogram Equalization (CLAHE) on the performances of the transfer learning models. To the best of the authors' knowledge, there is no similar study that investigates the use of the aforementioned Histogram Equalization techniques on datasets that use transfer learning in this task. Therefore, the main contributions of this study lie in the investigation of the use of these Histogram Equalization techniques when used on the original dataset.

In the following sections, an analysis of recent scholarly work done in the domain of detecting COVID-19 from lung scans is presented. Thereafter, a description of the methodology used in the implementation of this study is presented. Finally, an evaluation of the performances derived from the empirical experiments is discussed.

## 2. Related Work

The use of computer aided detection of diseases in medical images is a practice that dates back to the 1960s [13] and has shown consistent progress ever since, with many papers reporting highly accurate diagnosis of a range of conditions, such as breast cancer [14], osteoporosis, and cardiac disease [15]. The detection frameworks usually make use of ultrasound scans, x-ray images, and CT scans.

While the higher availability and lower cost of ultrasound and x-ray machines allow for more accessibility to patients, CT is generally considered to be the gold standard of radiological imaging tools, due to the high resolution and contrast images. These images can be improved through the use of certain Histogram Equalization techniques [16]. Recent work done to create computer vision tools that are able to detect COVID-19 from lung scans has often made use of deep learning tools. In this literature review, we present an overview of recent related studies to this topic.

Jaiswal et al. [10] investigated the performance of a DenseNet-201 architecture in detecting the presence of COVID-19 from CT lung scans. The use of a DenseNet-201 allows for the extraction of complex features for classification due to the 201-layer depth of the network, while avoiding the vanishing gradient problem. This problem occurs when the gradients from where the loss function is calculated shrink to zero after several applications of the chain rule. This results in the weights never updating their values and, therefore, no learning taking place. The DenseNet-201 solves this problem through the use of skip connections from initial filter layers to the ones found later. These skip connections allow for the gradients being able to flow back directly from the deeper layers of the convolutional base to the initial layers. In this paper, the DenseNet-201 base is further combined with an artificial neural network for classification, which consists of two hidden layers, made up of made up of 128 and 64 nodes, respectively, with ReLU activation functions, and a 2-node softmax output layer. The dataset used is the SARS-CoV-2 dataset. The paper reports an accuracy of 97% from the DenseNet-201, which slightly outperforms the VGG-16 and ResNet-152-V2, also used in the study, which show accuracies of 96% and 95%, respectively.

A recently published article by Marques et al. [12] investigated the use of the EfficientNet-B4 architecture in differentiating between x-ray lung scans that either have COVID-19, have general community acquired pneumonia, or are healthy. The EfficientNet architecture uses a process of uniformly scaling the networks width, depth, and resolution with a set of fixed scaling coefficients. It uses a principled way of scaling the width, depth, and resolution of the network to increase the receptive field for the extraction of more complex features from an image by maintaining a certain ratio to which each of those components is scaled. The EfficientNet-B4 architecture is a relatively newly developed architecture and, to the authors' knowledge, has had very little research done in evaluating its performance in this task, including any comparative evaluations on it against other transfer learning architectures. The EfficientNet-B4 architecture was chosen over the other EfficientNet architectures due to its 19 million parameters, which allowed for a degree of computational feasibility in this task, according to the authors. The 19 million parameters are a great deal less than the many of the other architectures investigated for this task in other literature; however, this paper reports that the EfficientNet-B4 shows impressive performances, with an accuracy, precision, recall, and F1-score of 96.7%, 97.54%, 96.69%, and 99.62%, respectively. This study was evaluated using a 10-fold validation process, and the architecture was not compared against other architectures. A three-hidden-layer artificial neural network with a three-node softmax output layer is attached to the convolutional base, and a 30% dropout rate is used to avoid overfitting.

In a paper by Islam et al. [17], an ensemble CNN-RNN architecture is proposed that investigates and compares the use of a VGG-19, DenseNet121, InceptionV3, and InceptionResNetV2 convolutional base, used for segmentation and feature extraction in combination with a recurrent neural network (RNN) classifier. The study aimed to create a framework that is capable of distinguishing between COVID-19 lung scans, community acquired pneumonia lung scans, and healthy lung scans. The paper describes the methodology of creating this ensemble model to be exactly the same as the methodology used when using a standard fully connected artificial neural network (ANN) for classification, with the only difference being the use of the RNN in place of an ANN for classification. In this study, a dataset that combines x-ray images from seven different sources, which include the Italian Society of Radiology, the Radiopaedia, and the Figshare data repository is used. The use of images from different sources may suggest an attempt in building a model that has a high degree of generalizability and does not overfit on a specific dataset. The combined dataset contains a total of 6939 samples, 1850 of which are COVID-19-positive lung scans, 1851 of which are community acquired pneumonia lung scans, and 1850 of which are healthy lung scans, which can be considered an equally distributed dataset. The study shows that the VGG-19-RNN architecture outperforms all other architectures, with 99.9 percent accuracy, 99.9% ROC-AUC, 99.8% recall, and 99.8% F1-score. This study represents one of the few ensemble architecture studies that incorporate transfer learning and is a definite potential avenue for future research, across newly developed transfer learning models.

Makris et al. [11] evaluated the use of several transfer learning frameworks for the differentiation between x-ray images of COVID-19 infected lung scans, lung scans infected with community acquired pneumonia, and healthy lung scans. The study compared the VGG-16, VGG-19, MobileNet-V2, InceptionV3, XCeption, InceptionResNet-V2, DenseNet-201, ResNet-152-V2, and NASNet-Large architectures and showed the VGG-16 and VGG-19 architectures to be the highest performing architectures, with overall accuracies of 95.88% and 95.03%, respectively, while the MobileNet-V2 and DenseNet-201 showed the lowest accuracies of 40% and 38%, respectively. The VGG-16 and VGG-19 also showed the highest recalls of 96% and 92%, respectively, while the MobileNet-V2 and DenseNet-201 showed the lowest recalls of 12% and 5%, respectively. The low performances of the MobileNet-V2 and DenseNet-201 models do not seem to reflect findings in other papers, which may be attributed to the use of different conditions under which the experiments were conducted, such as the use of 35 epochs to train the models, a number of epochs significantly lower than what is seen in other literature. The study uses a publicly available dataset, created by Dr. Joseph Cohen [18]. The paper does mention that while the DenseNet-201 and MobileNet-V2 architectures show lower accuracies than the highest performing models, the VGG-16 and VGG-19, they both outperformed the VGG-16 and VGG-19 in terms of precision and specificity. This may suggest that the use of the former models, which show lower accuracies, actually are more capable models if the goal of the task was to ensure that confirmed negative patients are accurately identified as negative. These metrics however are not the most important one. A higher importance to the recall metric is more valid in this task as a framework that has a greater capability of classifying a confirmed COVID-19-positive lung scan as COVID-19 positive would be more beneficial than a framework that is more capable of classifying a confirmed COVID-19-negative lung scan as negative. This is due to the fact that the recall or sensitivity of a test being higher means that, after testing, there is a lower chance of COVID-19-positive patients being reintroduced into society, having being tested as negative, and spreading the virus further.

## 3. Methods and Techniques

**Figure 1:**
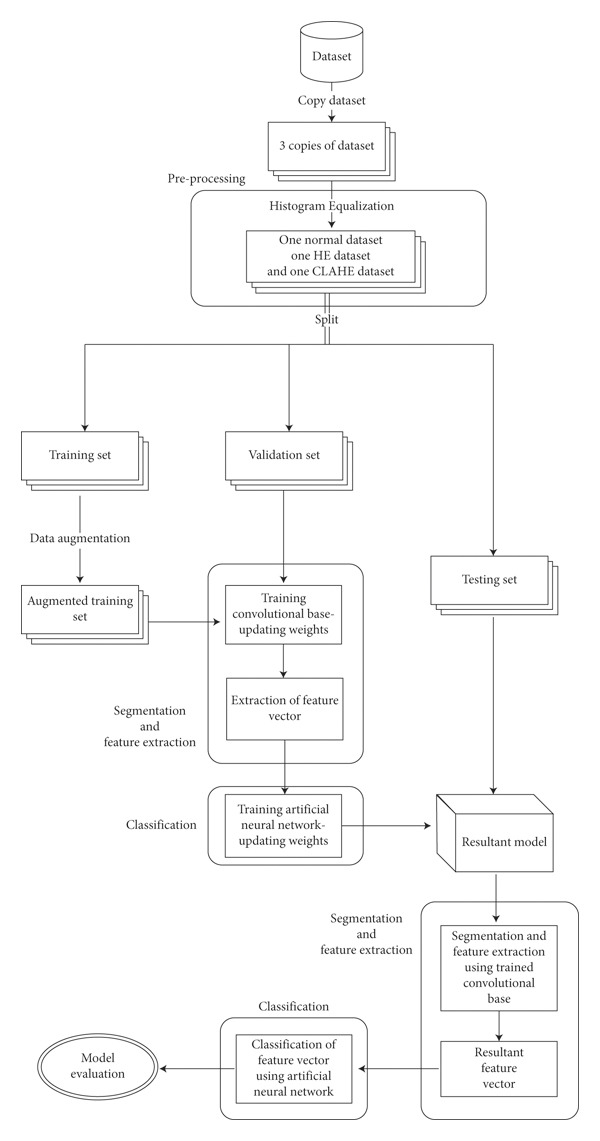
Proposed model for COVID-19 detection.

The proposed methodology follows a standard image processing pipeline of data acquisition, preprocessing, segmentation, feature extraction, and classification. The flowchart in Figure 1 gives an overview of the proposed model.

The initial step acquires the original dataset and two more copies, then using Histogram Equalization (HE) on one copy, and Contrast Limited Adaptive Histogram Equalization (CLAHE) on another, thereby creating 3 datasets.

From this point, each dataset is split into a training, validation, and testing set with the ratio 60 : 20 : 20. This stage also uses of data augmentation on the training sets, which creates dummy data, and allows for the training of models which are less prone to a phenomenon known as overfitting.

Segmentation and feature extraction are automatic processes, done in the following stage, by the convolutional bases of our transfer learning models, while the classification stage is carried out using a fully connected artificial neural network, which consists of a single 256-node hidden layer using the ReLU activation function, and a two-node softmax output layer. The entire process is discussed in more detail in the following sections.

### 3.1. Data Acquisition

**Figure 2:**
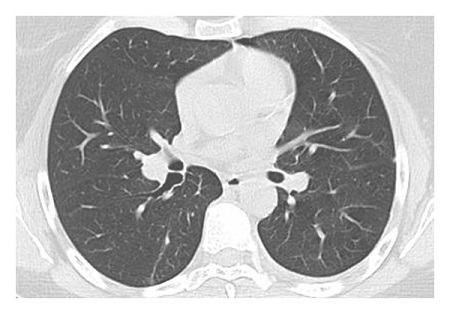
High quality COVID-19 negative slice.

**Figure 3:**
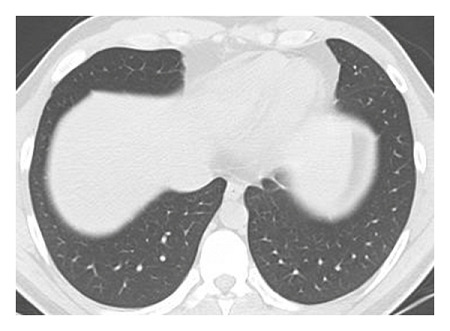
Obfuscated COVID-19 negative slice.

**Figure 4:**
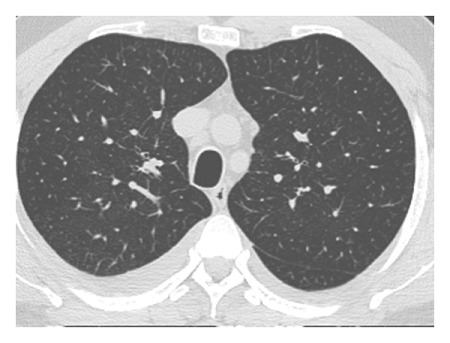
High quality COVID-19 positive slice.

**Figure 5:**
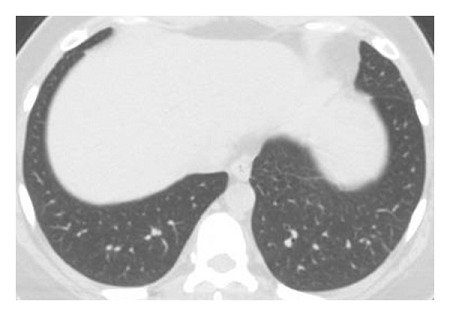
Obfuscated COVID-19 positive slice.

The dataset used for in this study is the SARS-CoV-2 CT scan dataset [19]. It consists of 2482 images obtained from patients located in São Paulo Brazil and is split into 1252 COVID-19-positive and 1230 COVID-19-negative images. The images found in this dataset show a relatively high degree of consistency and quality, with all images having the same dimensions and orientations, and no presence of Moire patterns or clinical markings, which are often found in images from other datasets. The dataset was specifically created with the intention of encouraging research into artificial intelligence methods that would be able to detect the presence or absence of the virus within an individual through an analysis of their CT scans. Some of the images found in the dataset may be seen in Figures 2–5.

### 3.2. Histogram Equalization and Contrast Limited Adaptive Histogram Equalization

After the acquisition stage, the original dataset is subjected to two different forms of Histogram Equalization, namely, the standard global version, which will be referred to as Histogram Equalization (HE), as well as an adaptive version, which will be referred to as Contrast Limited Adaptive Histogram Equalization (CLAHE). The purpose of Histogram Equalization is to effectively spread out the most frequent intensity values, i.e., stretch out the intensity range of the image, and in doing so create images of increased contrast. The increase in contrast allows for the presence of certain features to be enhanced and subsequently improve the performance in feature extraction.

Standard Histogram Equalization (HE) uses the same transformation derived from the image histogram to transform all pixels. This global equalization performs optimally when the distribution of pixel values is similar throughout the image. However, when the image contains regions that are significantly lighter or darker than most of the image, the contrast in those regions will not be sufficiently enhanced.

This problem is compensated for by Contrast Limited Adaptive Histogram Equalization (CLAHE). CLAHE transforms each pixel using a transformation function derived from a neighborhood region. Each pixel is transformed based on the histogram of a square, subregion of the image surrounding the pixel. This local equalization uses the exact same transformation function as the ‘global equalization' used in standard Histogram Equalization; however, it is done iteratively across distinct subsections of the image. The contrast limitation principle in CLAHE also prevents the overamplification of noise in relatively homogeneous regions of an image. This separates it from the standard Adaptive Histogram Equalization technique that does not use contrast limitation, which often sees the overamplification of noise in homogeneous regions.

**Figure 6:**
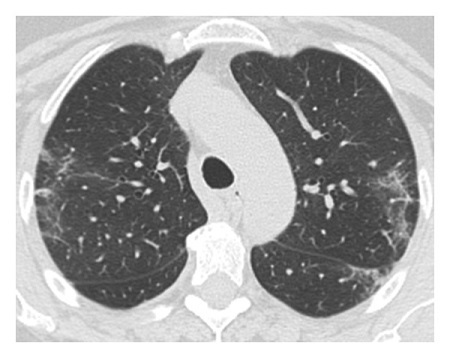
Unprocessed lung scan.

**Figure 7:**
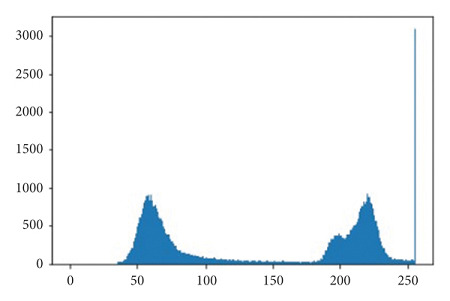
Unprocessed lung scan histogram.

**Figure 8:**
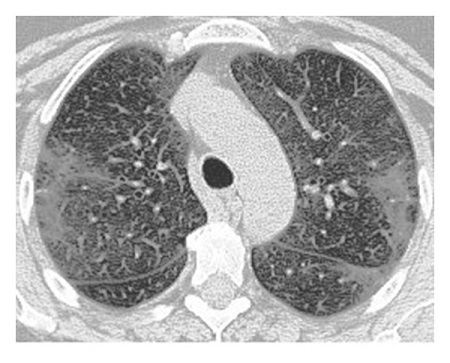
HE lung scan.

**Figure 9:**
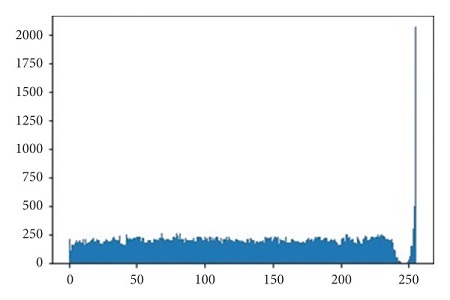
HE lung scan histogram.

**Figure 10:**
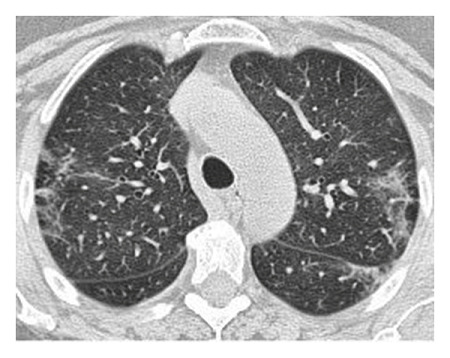
CLAHE lung scan.

**Figure 11:**
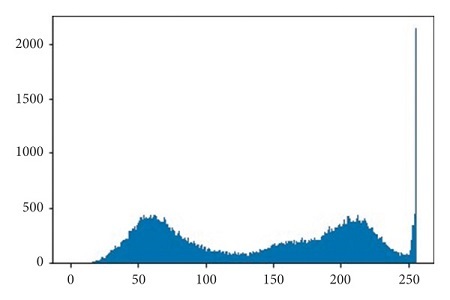
CLAHE lung scan histogram.

The illustrations of the use of both Histogram Equalization techniques on the lung scans are shown in Figures 6–11, with the images' corresponding histograms.

The figures show a large increase in finer details when standard Histogram Equalization is applied to the original image, however, a slight decrease in the area of larger artifacts within the original image, as well as what can be considered as an enhancement of background noise. The use of Contrast Limited Adaptive Histogram Equalization shows a great enhancement to the finer details of the image, while still preserving the area and brightness of larger artifacts. The number of distinct artifacts visible is less than the image that uses standard Histogram Equalization; however, there is also less degree of background noise than the image that uses standard Histogram Equalization.

### 3.3. Data Augmentation

**Figure 12:**
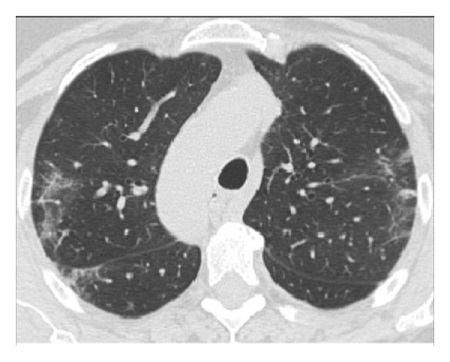
Horizontally flipped lung scan.

**Figure 13:**
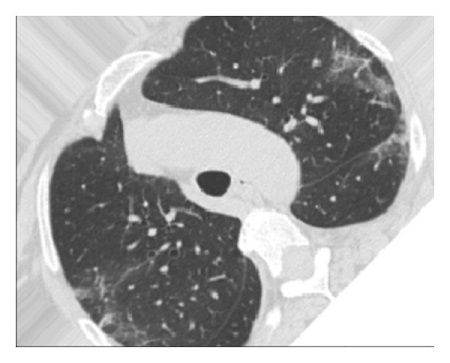
Negatively rotated lung scan.

**Figure 14:**
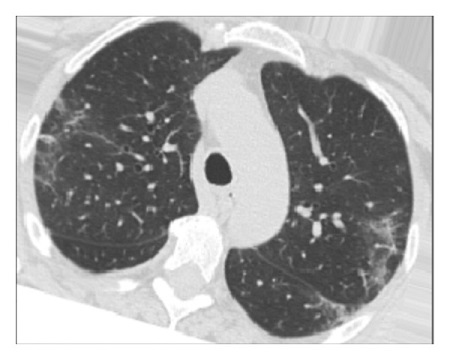
Positively rotated lung scan.

CNN models have been proven to perform extremely well on many computer vision tasks; however, they require a large amount of training data to avoid overfitting [20, 21]. Overfitting refers to the phenomenon where a machine learning model learns a function with a high degree of variance, such that the model perfectly models the training data, but has a low degree of generalizability. Unfortunately, in many cases, the ability to access large amounts of data for the optimal training of a CNN is not possible. An example can be seen when a new disease emerges, and we initially have a small amount of radiological images to train a model. Data augmentation comprises a variety of techniques that enhance the size and quality of training datasets, such that better deep learning models can be built using them. The use of these techniques creates “dummy data” which differ from the original data, in that it has been subjected to various rotations, width shifts, height shifts, and zooming. The use of these augmentation techniques also allows for the training of models that are more invariant to the range of orientations and configurations that different radiologists may use when inputting images to be classified. This study performs data augmentation by using the Python Image Data Generator library, to create images that see a rotation change of within a range of −40 to 40 degrees to the original images, a width shift, height shift, shear range, and zoom range of within 20 percent of the original image, and images that are flipped over the horizontal axis. Examples of the augmented images can be seen in Figures 12–14.

In the large scale application of a COVID-19 detection framework, the base machine learning model used for classification needs to be able to handle inputs that differ from the original training set, due to the fact that different radiologist inputting these images may decide to input images that do not perfectly match the orientations of the training set images. With this considered, a model that has a significant degree of generalizability is necessary, and it is key that a model used for this task performs data augmentation on its training data.

### 3.4. Experimental Setup

The architectures considered for this experiment are the ResNet-101, VGG-19, DenseNet-201, EfficientNet-B4, and MobileNet-V2 architectures. These architectures are reported to be amongst the best performing architectures in literature revolving around this topic [9–12], and the consideration of them in this experiment made sense due the high probability of them being able to produce a high performing framework for this task. The experiments across models are performed using the original, pretrained bases of the transfer learning models, to which a single, fully connected hidden layer, made up of 256 ReLU activated neurons is attached to the flattening layer of the convolutional base. This hidden layer is attached to a two-node softmax output layer, which gives a probabilistic output of a lung scan belonging to the COVID-19 positive or negative class. The convolutional bases perform automatic feature extraction, through the process of extracting feature maps from the original images, using the convolutional layers, and then converting these feature maps into a latent representation for classification by the fully connected artificial neural network.

The training process involves the learning of new weights for the convolutional base and artificial neural network classifier over time. This learning process allows for the convolutional base to extract features from the input image more optimally and allows for those features to be processed and classified more accurately by the artificial neural network. The training process uses the categorical cross entropy loss function and RMSprop optimizer to iteratively update the weights over time. The updating of the weights ideally reduces the loss over time. This decrease in loss correlates to a model that produces a small number of misclassified instances, which is ideal for a classification problem.

Each model is trained over 200 epochs, and uses a learning rate of 2*x*10^−5^.

**Figure 15:**
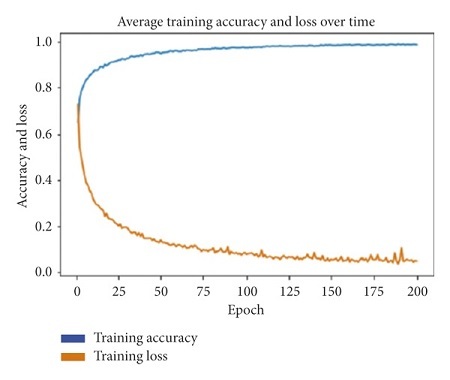
Average training accuracy vs. average loss calculated across all models.

The decision to use these hyperparameters was arrived at through a process of manual hyperparameter tuning, which started with the use of 50 epochs and a learning rate of 0.001 [22, 23], and increased from that point, until a point where the models showed a peak performance. Each model was trained using a training and validation batch size of 20, and tested using a batch size of 15.  Figure 15 shows the average training accuracy and loss calculated over all 15 experiments done. The graph shows an increase in training accuracy over time, which directly correlates with the decrease in loss over time.

The experiments are conducted on 64-bit Windows 10 desktop machine, which comprises a 16 GB DDR4 RAM and Intel i-7-7700 processor, running at 3.60 GHz. The study is conducted in a Jupyter Notebook environment and uses the Python language. A significant portion of the code makes use of the TensorFlow and sklearn libraries, which assist in model development, training, and evaluation.

### 3.5. Results and Discussion

**Figure 16:**
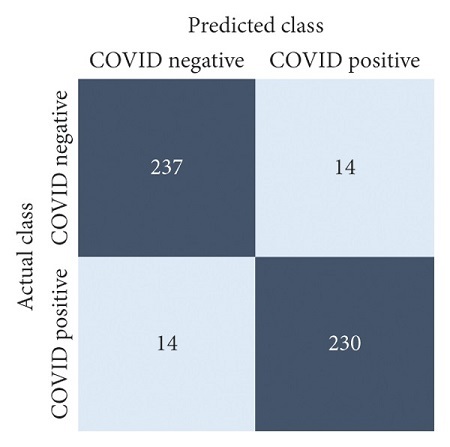
DenseNet-201: no equalization.

**Figure 17:**
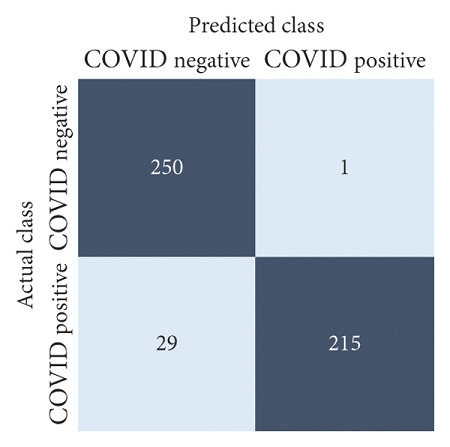
DenseNet-201: HE.

**Figure 18:**
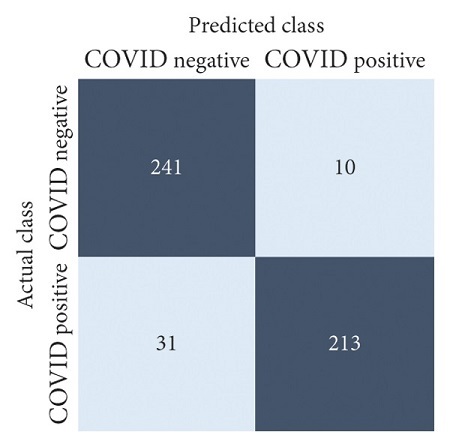
DenseNet-201: CLAHE.

**Figure 19:**
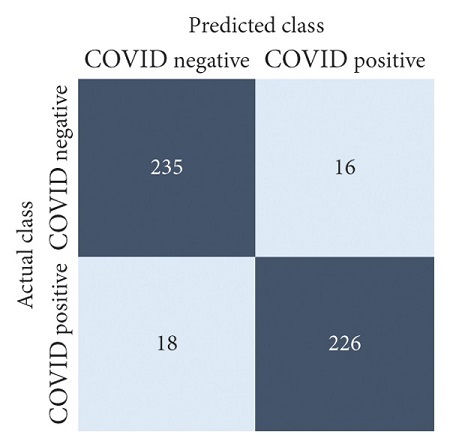
ResNet-101: no equalization.

**Figure 20:**
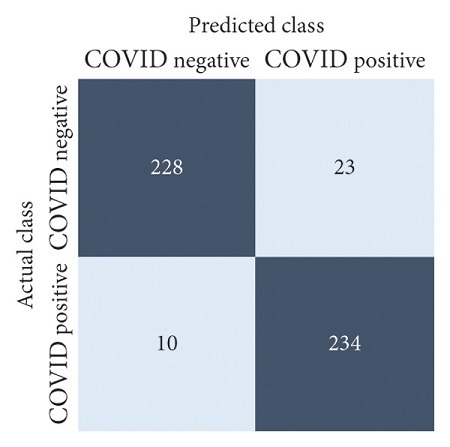
ResNet-101: HE.

**Figure 21:**
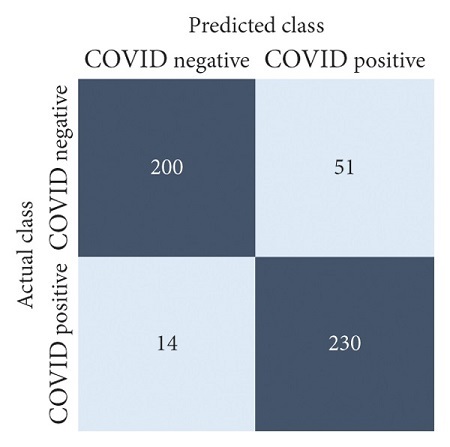
ResNet-101: CLAHE.

**Figure 22:**
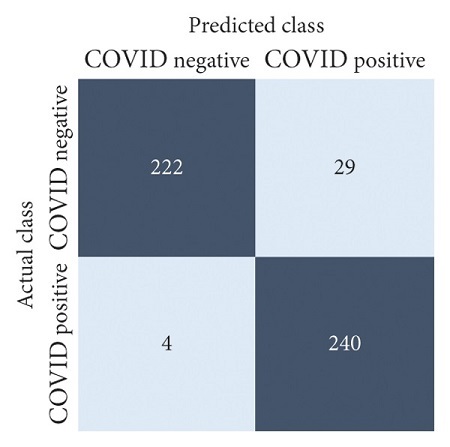
VGG-19: no equalization.

**Figure 23:**
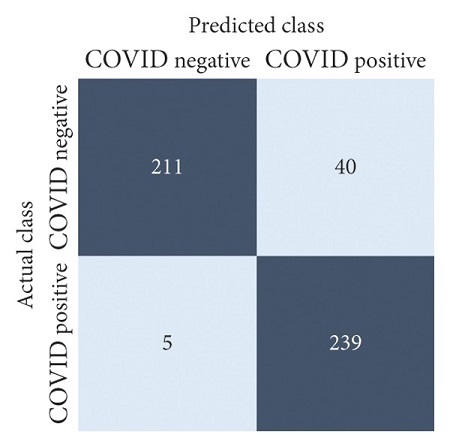
VGG-19: HE.

**Figure 24:**
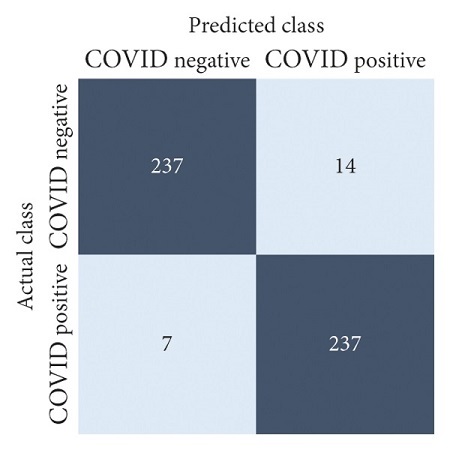
VGG-19: CLAHE.

**Figure 25:**
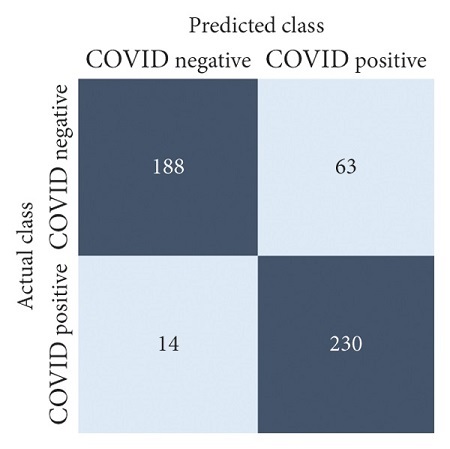
EfficientNet-B4: no equalization.

**Figure 26:**
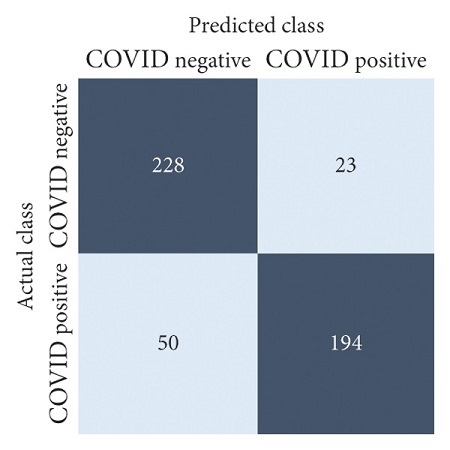
EfficientNet-B4: HE.

**Figure 27:**
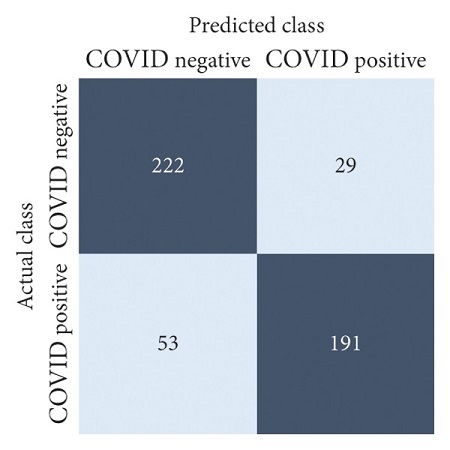
EfficientNet-B4: CLAHE.

**Figure 28:**
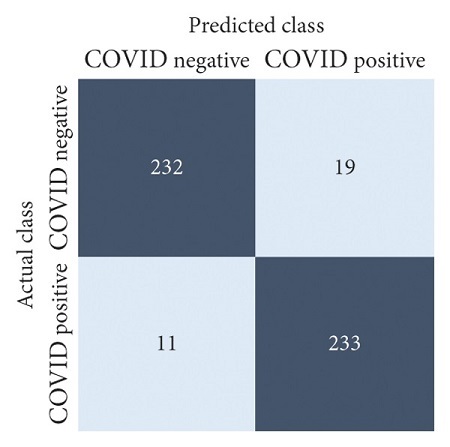
MobileNet-V2: no equalization.

**Figure 29:**
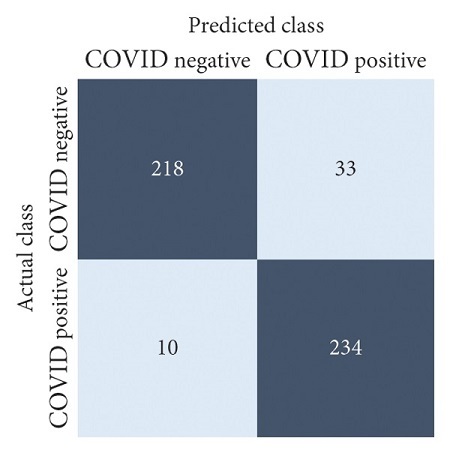
MobileNet-V2: HE.

**Figure 30:**
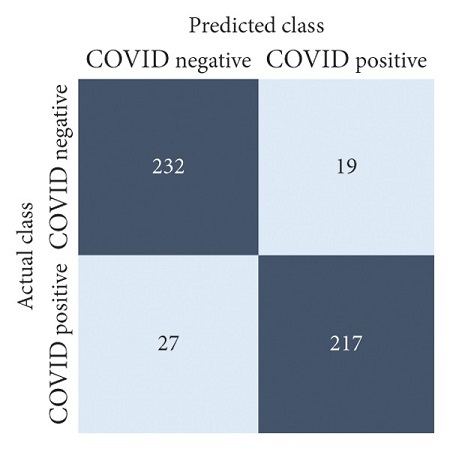
MobileNet-V2: CLAHE.

The performances of all experiments are evaluated by using a series of confusion matrix-based performance metrics. These values reflect the performances of the trained model on the testing set. The metrics evaluated are accuracy, precision, recall, F1-score, specificity, and ROC-AUC. The confusion matrices used to evaluate the classifiers are shown in Figures 16–30, with true positives (TP) representing the COVID-19-positive lung scans that are correctly classified as positive, true negatives (TN) representing COVID-19-negative lung scans that are correctly classified as negative, false positives (FP) representing COVID-19-negative lung scans that are incorrectly classified as positive, and false negatives (FN) representing COVID-19-positive lung scans being incorrectly classified as negative. These metrics are explained in greater detail in Sections 3.5.1to 3.5.6.

#### 3.5.1. Accuracy

It is a measure that indicates the ratio of all the correctly recognized cases to the overall number of cases. While this metric generally gives a decent reflection of the classifier, it may not reflect a classifier's true performance in a scenario where there is an uneven class distribution. Accuracy can be computed using the following formula:

(1) $$\begin{array}{c} \text{accuracy}=\frac{\text{TP}+\text{TN}}{\text{TP}+\text{TN}+\text{FP}+\text{FN}}. \end{array}$$

#### 3.5.2. Precision

It is the ratio of all correctly classified positive instances by a model to the overall number of positive classifications by a model. A low precision indicates that a model suffers from a large number of false positives. This measure, along with recall, F1-score, and specificity, is more capable of handling class distribution issues. Precision can be computed using the following formula:

(2) $$\begin{array}{c} \text{precision}=\frac{\text{TP}}{\text{TP}+\text{FP}}. \end{array}$$

#### 3.5.3. Recall

Also called sensitivity, it is the ratio of all correctly classified positive instances by a model to the overall number of true positive instances being tested. Recall can be computed using the following formula:

(3) $$\begin{array}{c} \text{recall}=\frac{\text{TP}}{\text{TP}+\text{FN}}. \end{array}$$

#### 3.5.4. F1-Score

It is used as the harmonic mean of precision and recall. A high F1-score is obtained when there is some sort of balance between precision and recall. If the F1-score is not very high, this may be an indication of one of these metrics being improved at the expense of the other. F1-score can be calculated using the following formula:

(4) $$\begin{array}{c} F1-\text{score}=\frac{2\times \text{precision}\times \text{recall}}{\text{precision}+\text{recall}}. \end{array}$$

#### 3.5.5. Specificity

It is the ratio of correctly classified negative instances by a model to the overall number of true negative instances being tested. Specificity can be calculated using the following formula:

(5) $$\begin{array}{c} \text{specificity}=\frac{\text{TN}}{\text{TN}+\text{FP}}. \end{array}$$

#### 3.5.6. Area under the Receiver Operating Characteristic Curve

**Figure 31:**
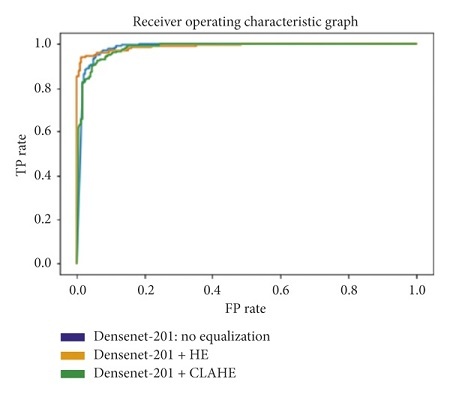
ROC graph: DenseNet-201.

**Figure 32:**
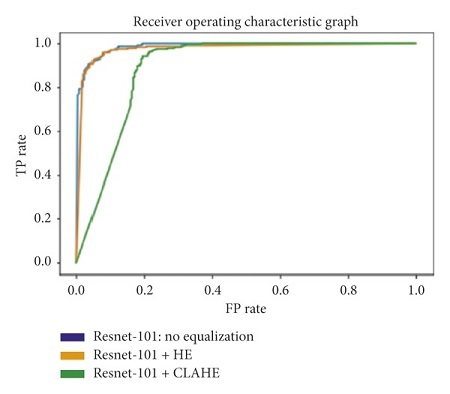
ROC graph: ResNet-101.

**Figure 33:**
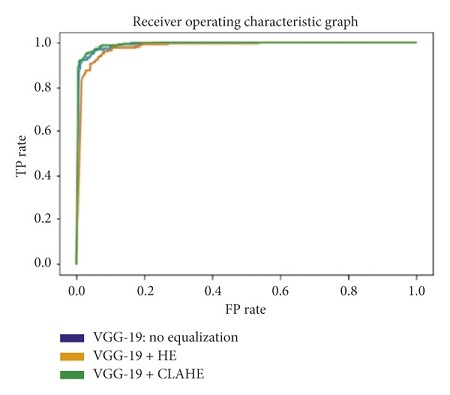
ROC graph: VGG-19.

**Figure 34:**
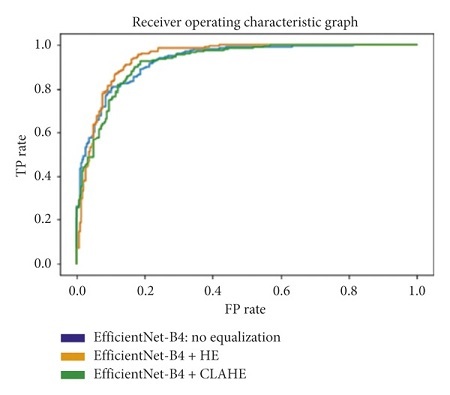
ROC graph: EfficientNet-B4.

**Figure 35:**
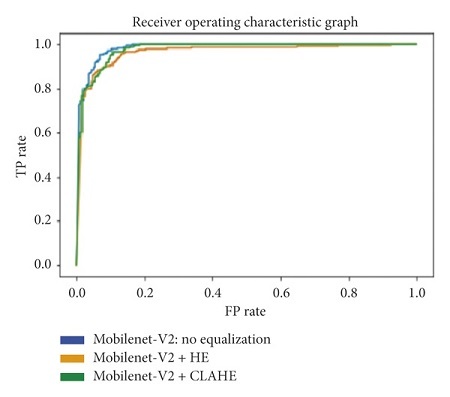
ROC graph: MobileNet-V2.

The receiver operating characteristic curve (ROC curve) is a graph that shows the performance of a classification model at all classification thresholds. The curve plots the true positive rate (recall) against the false positive rate (1−specificity). Lowering the classification threshold classifies more items as positive, which increases both the false positive and true positive rates. The use of the area under the receiver operating characteristic curve (ROC-AUC) provides an aggregate measure of performances across all possible classification thresholds. In this task, a higher ROC-AUC implies that a model is better at predicting true negative lung scans as negatives, and true positive lung scans as positives. The ROC curves for each transfer learning architecture, used on the original, Histogram Equalized, and Contrast Limited Adaptive Histogram Equalized datasets, are shown in Figures 31–35.

For the purposes of this study, the most important metric to consider is the recall [11].

While the other metrics are also very important, a model that shows a low recall indicates that that particular model is susceptible to testing a patient as COVID-19 negative, when they are actually positive. This may result in a large number of COVID-19 positive patients being reintroduced into society, completely unaware of the fact that they are infected, contributing to the spread of the virus. It may also mean that a patient does seek medical treatment for their infection, which may lead to death. Another metric also very important is the ROC-AUC, as it gives a good overall probability of a model being able to predict a true negative as negative, and a true positive as positive. The results of our experiments can be seen in Tables 1–5.

From the results in Tables 1–5, the VGG-19 architecture achieves the highest recalls across all three datasets. Given that the difference in the recall is not significant enough to show an experiment with the best results, the ROC-AUC, accuracy, and F1-score are used to determine it. The VGG-19, trained and tested on a dataset that uses Contrast Limited Adaptive Histogram Equalization, achieved the optimum results. This particular combination shows an accuracy, recall, F1-score, and ROC-AUC of 95.75%, 97.13%, 95.75%, and 99.30%, respectively.

While this model shows the highest performances on those specific metrics, its specificity of 94.42% is not as good the specificity of 99.60% shown by a MobileNet-V2 architecture, trained on a Histogram Equalized Dataset. Given a different task, the consideration of choosing a model's specificity as the most important metric may have been more appropriate. This task, however, would not benefit as much from a model that is more capable of classifying true negatives as negative, but would benefit more from a model that has high capability of classifying true positives as positive.

The results of the experiment show no conclusive proof that Histogram Equalization and Contrast Limited Adaptive Histogram Equalization have any significant impact on the overall performance across all models, with different models showing varying performances when different equalization techniques are used on their datasets.

Versaci et al. [24] proposed a fuzzy technique for adaptive gray-level image contrast enhancement. The proposed technique enables high contrast enhancement in all the images with performance that is fully comparable with that obtained by three more sophisticated fuzzy techniques and by standard Histogram Equalization. Furthermore, Versaci and Morabito [25] presented a new fuzzy edge detector based on both fuzzy divergence and fuzzy entropy minimization for the thresholding substep in gray-scale images. The fuzziness content of each image has been quantified by specific indices formulated in terms of fuzzy divergence. Therefore, these are alternative techniques for future work in this research work.

### 3.6. Literature Comparison

A comparative analysis of other studies investigating the use of transfer learning in the detection of COVID-19 in lung scans is presented in this section. A tabular comparison of the performances shown in the various papers is shown in Table 6.

Apostolopoulos et al. [26] reported the highest accuracies in detecting COVID-19 in lung CT scans when using the MobileNet-V2 and VGG-19 architectures. This study was done using a dataset obtained from various sources. The dataset contained 224 COVID-19-positive lung scans and 504 COVID-19-negative lung scans. The unevenly distributed dataset may have contributed to some skewing of the accuracy.

Markris et al. [11] reported the use of a VGG-16 producing the highest accuracies and recalls in a study that used a publicly available dataset on Kaggle, created by Dr Joseph Cohen [18]. The study uses a dropout of 50%, with the models being trained over 35 epochs.

Marques et al. [12] investigated the use of the EfficientNet-B4 architecture. The study uses a 3-layer artificial neural network, which may have resulted in higher accuracies than that which may have come from using fewer layers, as seen in other studies. A dropout of 30% is used in this study.

Jaiswal et al. [10] investigated the use of the DenseNet-201 architecture. The study uses the SARS-CoV-2 CT scan dataset [19], mentioned earlier in this paper. The model uses a 2-hidden-layer artificial neural network, which may contribute to an increase in accuracy that does not reflect the true performance of the transfer learning convolutional base. The only metric reported in this study is the accuracy of the trained model.

Islam et al. [17] investigated the use of CNN-RNN ensemble architectures in this task. The use of the VGG-19 architecture produces the highest accuracies and recalls. A dataset that combines images from various sources is used.

Das et al. [27] reported the use of the XCeption architecture producing the highest accuracies in this task. The dataset used is obtained from a publicly available dataset, created by Ozturk et al. [28].

Ardakani et al. [29] reported the use of the ResNet-101 architecture outperforming 9 other architectures, including the ResNet-50. The study uses a dataset obtained manually from patients in a hospital in the Toshiba Medical Center in Japan. The study also compared the results of the experiments against human experts. The human experts are reported to show accuracies and recalls of 86.27% and 89.21%, respectively.

### 3.7. Conclusion and Future Work

In this paper, an investigation into 5 different transfer learning architectures is conducted, to determine their performances when tasked with detecting COVID-19 in lung CT scans. In addition, the study also evaluates the impact of using standard Histogram Equalization and Contrast Limited Adaptive Histogram Equalization on the the lung scans. The final results show that the VGG-19 architecture, combined with a dataset that uses Contrast Limited Adaptive Histogram Equalization, showed the best overall performance, with an accuracy of 95.75%, recall of 97.13%, F1-score of 95.75, and ROC-AUC of 99.30%. The final results of our study do not give a definitive answer on whether or not the Histogram Equalization techniques used actually have an overall effect on performance across all models, with some architectures showing higher performances with Histogram Equalization, and some showing higher accuracies without Histogram Equalization.

Given the high demands on COVID-19 testing, as well as the availability of CT scans in most medical institutions, the use of a framework, based on a high performing architecture like the VGG-19 described above, can pose an alternative to the currently used testing methods and help reduce the bottleneck on those resources.

Future work on this topic should include the investigation of automatic hyperparameter optimization techniques on transfer learning models, as well as the development of transfer learning-based frameworks that allow for the processing of 3D CT scans.

## Figures and Tables

**Table 1:** DenseNet-201 performances.

Model	Accuracy %	Precision %	Recall %	F1-score %	Specificity %	ROC-AUC %
DenseNet-201: no equalization	94.34	94.26	94.26	94.26	92.43	98.39
DenseNet-201 + HE	93.93	99.53	88.11	93.47	86.85	99.02
DenseNet-201 + CLAHE	91.71	95.51	87.29	91.22	92.43	98.22

**Table 2:** ResNet-101 performances.

Model	Accuracy %	Precision %	Recall %	F1-score %	Specificity %	ROC-AUC %
ResNet-101: no equalization	93.13	93.38	92.62	93.00	93.62	98.65
ResNet-101 + HE	93.33	91.05	95.90	93.41	90.83	97.67
ResNet-101 + CLAHE	86.86	81.85	94.26	87.61	79.68	88.91

**Table 3:** VGG-19 performances.

Model	Accuracy %	Precision %	Recall %	F1-score %	Specificity %	ROC-AUC %
VGG-19: no equalization	93.33	89.21	98.36	93.56	88.44	98.99
VGG-19 + HE	90.90	85.66	97.95	91.39	84.06	98.01
VGG-19 + CLAHE	95.75	94.42	97.13	95.75	94.42	99.30

**Table 4:** EfficientNet-B4 performances.

Model	Accuracy %	Precision %	Recall %	F1-score %	Specificity %	ROC-AUC %
EfficientNet-B4: no equalization	84.44	78.49	94.26	85.66	74.90	93.15
EfficientNet-B4 + HE	85.25	89.40	79.50	84.16	90.83	94.29
EfficientNet-B4 + CLAHE	83.43	86.81	78.27	82.32	88.44	92.58

**Table 5:** MobileNet-V2 performances.

Model	Accuracy %	Precision %	Recall %	F1-score %	Specificity %	ROC-AUC %
MobileNet-V2: no equalization	93.93	92.46	95.49	93.95	94.42	98.30
MobileNet-V2 + HE	91.31	87.64	95.90	91.58	99.60	96.45
MobileNet-V2 + CLAHE	90.70	91.94	88.93	90.41	96.01	97.63

**Table 6:** Literature comparison of classification accuracies and recalls.

Model	Accuracy %	Recall %
MobileNet-v2 [26]	97.40	99.10
VGG-19 [26]	98.75	92.85
VGG-16 [11]	95.88	96.00
EfficientNet-B4 [12]	96.70	96.69
DenseNet-201 [10]	97.00	—
VGG-19 + RNN [17]	99.9	99.8
XCeption [27]	99.52	99.12
ResNet-101 [29]	99.51	100
VGG-19 + CLAHE (proposed)	**95.75**	**97.13**

## Data Availability

The data used to support the findings of this study are publicly available.
